# Microbial Monitoring of Surface Water in South Africa: An Overview

**DOI:** 10.3390/ijerph9082669

**Published:** 2012-07-30

**Authors:** Catherine D. Luyt, Roman Tandlich, Wilhelmine J. Muller, Brendan S. Wilhelmi

**Affiliations:** 1 Environmental Health and Biotechnology Research Group, Division of Pharmaceutical Chemistry, Faculty of Pharmacy, Rhodes University, P.O. Box 94, Grahamstown 6140, South Africa; Email: g04l4089@campus.ru.ac.za; 2 Unilever Centre for Environmental Water Quality, Institute for Water Research, Rhodes University, P.O. Box 94, Grahamstown 6140, South Africa; Email: nmuller@amatolawater.co.za; 3 Department of Biochemistry, Microbiology and Biotechnology, Rhodes University, P.O. Box 94, Grahamstown 6140, South Africa; Email: b.wilhelmi@ru.ac.za

**Keywords:** *E. coli*, national microbial monitoring programme, bifidobacteria, H_2_S strip test

## Abstract

Infrastructural problems force South African households to supplement their drinking water consumption from water resources of inadequate microbial quality. Microbial water quality monitoring is currently based on the Colilert^®^18 system which leads to rapidly available results. Using *Escherichia coli* as the indicator microorganism limits the influence of environmental sources on the reported results. The current system allows for understanding of long-term trends of microbial surface water quality and the related public health risks. However, rates of false positive for the Colilert^®^18-derived concentrations have been reported to range from 7.4% to 36.4%. At the same time, rates of false negative results vary from 3.5% to 12.5%; and the Colilert medium has been reported to provide for cultivation of only 56.8% of relevant strains. Identification of unknown sources of faecal contamination is not currently feasible. Based on literature review, calibration of the antibiotic-resistance spectra of *Escherichia coli* or the bifidobacterial tracking ratio should be investigated locally for potential implementation into the existing monitoring system. The current system could be too costly to implement in certain areas of South Africa where the modified H_2_S strip test might be used as a surrogate for the Colilert^®^18.

## 1. Introduction

Historically, the degree of development of drinking water infrastructure has varied significantly among different geographical areas in South Africa [1]. Prior to 1994, the majority of the black population was confined to homelands and townships with limited if any infrastructure [2]. This has resulted in the development of large settlements with no drinking water infrastructure. After 1994, the South African government started to address these issues and major improvements have been achieved. By 2002, 84.5% of the population had access to piped and tap water inside their homes or within 200 m of their residence from communal or neighbours’ taps/boreholes [3]. A coverage increase to 89.3% was achieved by 2010 [3]. This contributed to a decrease in the average risk of contracting diarrhoeal diseases among children under 5 years of age from 17.8% in 2002 to 11.2% in 2010 [4]. 

Around 65% of all economic activity is concentrated in the metropolitan areas of the Gauteng Province and the municipalities of Cape Town, eThekwini and Nelson Mandela Bay [5]. In 2010, 62% of the South African population lived in urban centres, likely the economic hubs, with an estimated growth rate of 1.2% per annum [6]. Based on the last percentage mentioned, South Africa is in the middle of the global interval for urbanisation [7]. Continuous migration into the most industrialised provinces such as Gauteng [8] has placed higher demand on service delivery and resources such as drinking water [5,9]. Some success in matching this increasing demand has been achieved as 96 to 98% of the South African population living in informal settlements had access to safe drinking water by 2010 [10].

Literature data presented so far shows that immense progress, in the delivery of drinking water and access to safe drinking water resources, has been achieved in the past two decades. However, problems are still rife. The total population of South Africa reached 44.8 million in 2001 and it was estimated at 47.9 million in 2007 [11], giving an increase of 6.5%. At the same time, the number of HIV/AIDS rose by 15% [8]. For a comparable time period, the number of deaths from the intestinal infectious diseases rose from 14,276 in 2000 to 37,398 in 2007 in South Africa [12], *i.e.*, indicating an increase of 61.8% in the rate of deaths due to the intestinal infectious diseases. Therefore South African citizens are at heightened risk of mortality from intestinal diseases over the past decade. Many of such diseases are waterborne despite improved access to safe drinking water.

The increased risk can’t be explained by population growth or the increase in the number of immunocompromised patients alone, who are often more susceptible to waterborne diseases than the general population [13]. Other factors must be involved such as inadequate sanitation and irregular access to safe drinking water [14]. The latter could be the result of infrastructural problems and interruptions in water supply. These include burst pipes at the household level [15], the unsatisfactory upkeep of communal taps [16] and inadequate operation of the water-treatment works by unskilled personnel leading to high levels of microbial contamination in drinking water [17]. In 2010, 47.6% of households in South Africa experienced interruptions of water supply and 36.2% of such outages lasted more than 15 days at a time [10].

Based on the above mentioned information, a considerable part of the population in South Africa is likely to intermittently rely on water resources of inadequate microbial quality to meet their demand for domestic use [17,18]. Domestic use includes water for drinking, laundry, cooking and personal hygiene [19] and the water resources in question include streams, springs, and rivers, *i.e.*, surface water resources. The public health risk is measured by the concentration of indicator organisms such as faecal coliforms [19] and *Escherichia coli* (*E. coli*) [20]. Raw water from these sources can meet the microbial criteria for domestic use after a minimum treatment, *i.e.*, boiling, addition of bleach or sand filtration [21]. Boiling can, however, be time-consuming at the household level and bleach is often too expensive for poor households [22]. Thus treatment is frequently not taking place before domestic use, which in turn increases the chances of a waterborne disease outbreak, e.g., from hepatitis E [23] and cholera [24,25]. Therefore monitoring of microbial quality of surface water in South Africa becomes an important tool in public health protection. This paper provides a critical review of the current system, along with an analysis of its advantages and drawbacks; and concludes with suggested improvements. 

## 2. Legislative Framework for the Microbial Quality Monitoring of Surface Water in South Africa

With respect to water resource management, South Africa is divided into 19 Water Management Areas (WMAs) [21,26]. The microbial water quality monitoring is the responsibility of various departments at all levels of government. The National Health Act of South Africa (NHA) defines the responsibilities of local and district municipalities by including water monitoring and environmental pollution monitoring among municipal services [27]. The National Water Act states in chapter 14 part 1 paragraph 137 that the Minister is responsible for setting up a monitoring system for microbial water quality of surface water among others [21,26]. Given the structure of the national government, the term “Minister” applied to the Minister of Water Affairs and Forestry up to the end of April 2009. Currently, this is the Minister of Water and Environmental Affairs, due to the re-arrangement of responsibilities within government areas in May 2009. The monitoring system of microbial water quality applies to the National Microbial Monitoring Programme for surface water (NMMP) [21] that is discussed in detail in the next section. 

## 3. National Microbial Monitoring Programme for Surface Water (NMMP)

The NMMP was developed based on research by du Preez *et al.* [28,29,30]. One of the aims of the NMMP was to create a central hub for microbial water quality data in South Africa [21]. The Department of Water Affairs in the Ministry of Water and Environmental Affairs (DWA) administers the programme, and also operates the national information system of microbial water quality—the Central Water Quality Database [21]. The DWA is responsible for the appointment of the national coordinator for the NMMP [21,31]. The appointment of an assistant national coordinator has been recommended, to guarantee running of the programme in the event of resignation of the national coordinator or during exceptional situations [21]. 

A regional coordinator is appointed in each of the WMAs to oversee the running of the NMMP. All coordinators are employees of DWA and their responsibilities include management of collection and analyses of water samples at the prioritised sampling sites once every 7 to 14 days [21,32]. Turbidity, pH and the concentration of *E. coli* are determined in all samples and data are communicated to the Central Water Quality database [21]. Periodical reports are written every two months by the regional coordinator on the main trends in the monitoring results. These reports are subsequently sent to the national coordinator who forwards them to the Ministry of Water and Environmental Affairs (MWEA), decision-makers in the DWA, the National Department of Health, relevant water resource authorities and any stakeholder that expresses interest [21].

There are 278 tertiary catchments in South Africa and sampling water in every one of the sites is a logistically challenging task. Therefore prioritisation is the only way to assess the status of the faecal contamination, given the finite financial resources that are available [33]. The priority sampling sites are generally selected by local staff under leadership of the regional NMMP co-ordinator [21]. During this process, several criteria are taken into account. The first one is the land uses that are most likely to function as point or non-point sources of faecal contamination of surface water. These include informal settlements in the vicinity of the water resources in question and agricultural activities, such as livestock watering [21]. The second criterion is the size of the population that is likely to come into contact with the water resource in question [21]. The last criterion accounts for the water uses in the area [21].

Prioritisation of catchments within a WMA starts with the collection of preliminary data. These data include identification of catchment areas that have experienced microbial water quality problems in the past, information about areas with high prevalence of waterborne diseases and areas in the catchment where the population is using untreated or partially treated surface water to meet domestic water demand [21]. The sources of information in the prioritisation process are the DWAF Water Supply and Sanitation Database, the Agricultural Research Council, the Regional Deputy Directors of DWA and the Regional Water Quality Managers and Water User Associations. In May 2009, the responsibility for the sanitation service delivery was transferred from the Department of Water and Environmental Affairs to the Department of Human Settlements at the national level of government [34]. Therefore relevant documents from this Department should also be consulted during prioritisation of new sampling sites. Once data collection is completed, the catchments fulfilling all of the following criteria are short-listed for ranking [21]. 

The ranking process is based on quantitative assessment of land and water uses in the catchment (areas) that were chosen using the preliminary information. The land use rating (*LUR*) and the water use rating (*WUR*) are then calculated using Equations (1,2): 


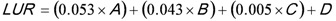
(1)


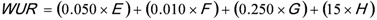
(2)

In Equation (1), *A* is the number of individuals that do not have access to sanitation infrastructure and *B* stands for the number of individuals with access to inadequate, poorly maintained or badly operated sanitation infrastructure. At the same time, *C* represents the mean density of the population and is also a measure of the rain-impermeable surfaces area. *D* is the number of the intensive livestock farming facilities that lack proper waste handling procedures and are located within the given catchment. In Equation (2), *E* describes the number of individuals who rely on untreated surface water to meet their domestic water/drinking water demand in the particular catchment. The term *F* in Equation (2) characterises the portion of the population in a given area that rely on surface water to meet their domestic water/drinking demand and use after partial water treatment. The highest possible number of people who come into full or partial contact with the surface water in a month is lumped into the term *G* in Equation (2). Finally, *H* stands for the number of hectares of land on which vegetables are grown and irrigated with the surface water being rated, in the same equation.

Once the values of *LUR* and *WUR* have been calculated, the overall area rating (*OAR*) is calculated using Equation (3):


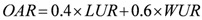
(3)

Murray *et al.* [21] recommended that the monitoring should automatically be conducted in areas with the *OAR* values of 200,000 and above. This value applies only to areas with high levels of faecal contamination in the given surface water resources and it can be lowered or increased depending on the logistical and financial capabilities or local monitoring needs of the WMA [35]. Once the selection of all sampling sites has been completed, monitoring commences. At the end of a given year, the health risks are evaluated using the concentration of *E. coli* and the water uses in the priority area. Such an evaluation is based on the calculation of the potential health risk index *PHRI* according to Equation (4):


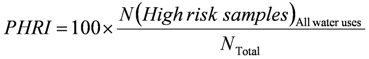
(4)

In Equation (4), *N*(High risk samples)_All water uses_ refers to the number of water samples taken at all sampling sites in a given priority area; and where the concentration of *E. coli* indicates a high health risk to the human population for all relevant water uses [21]. Then *N*_Total_ is the total number of samples taken over the period of a year at all selected sampling sites in a given priority area. The *PHRI* values below 30 indicate limited faecal contamination of the water resources in the particular priority area and limited risk to public health associated with a given water use. If the *PHRI* values range from 30 to 60, then the surface water resources suffer from medium level of faecal contamination in the particular priority area. Finally if the *PHRI* values are above 60 then a high level of faecal contamination of the water resources in the particular priority area can be inferred. This also infers a high level of public health risk if the surface water is used to meet domestic use demand.

Implementation of the NMMP has been rolled-out in phases to identify and eliminate as many problems as possible in the early stages of its operation [21]. For the successful functioning of the NMMP, sufficient capacity to carry out all of the required functions must exist in a given WMA [21]. The role of the different stakeholders should be clearly defined at the beginning of the programme’s implementation, preferably through the signing of legally binding contracts/agreements [21]. Coverage of the country’s territory in the context of the NMMP can be found at the respective DWA website [36].

## 4. Advantages of the Existing Microbial Water Quality Monitoring Framework

Faecal contamination detection requires that the indicator microorganism used is a member of the microflora of human and animal intestinal tract(s). It must be present in a water sample if faecal contamination is present [18]. With regard to detection of waterborne pathogens, the indicator microorganism must fulfill the following criteria: it must be present in a water sample when the pathogen in question is present, it must be absent when the pathogen is absent in a given water sample; and lastly the indicator microorganism must have similar survival characteristics in the environment to the pathogen in question [37]. The NMMP is based on the enumeration of *E. coli*. The rationale for using this organism is that the majority of faecal coliforms excreted in faeces of healthy humans and warm-blooded animals, such cattle and pigs are accounted for by *E. coli* [38].

At the same time, a clear relationship has been reported between the concentration of *E. coli* in a particular water sample and the probability of gastroenteritis symptoms in humans exposed to the water through drinking or full-contact recreation [39]. Therefore *E. coli* fulfils the above criteria for an indicator organism. Furthermore soils are the only environmental source of *E. coli* [40,41]. As a result, the influence of environmental contributions to the measured concentrations of *E. coli* at a given sampling site will be negligible, *i.e.*, providing a clear understanding of the faecal contamination at a given sampling site [42]. Similar conclusions can be drawn about the public health risks from faecal contamination of water resources.

Enumeration of *E. coli* for the NMMP is done using the Colilert^®^18 system. It is a most-probable number (MPN) technique which provides quantitative information on faecal contamination after 18 hours of incubation [21,43]. This period is much shorter than 24 to 48 hours required in other routine tests [18,19]. Thus the choice of this system provides the fastest turnaround time for results to be available to the regulatory authorities and stakeholders through the NMMP. The water quality guidelines for domestic use and drinking water regulations require that the concentration of *E. coli* or faecal coliforms is below 0 CFUs or cells/100 cm^3^ [19,20]. The Colilert^®^18 system does have this sensitivity and therefore can be deemed suitable for rapid and sensitive detection of the faecal contamination of surface water [43]. On the other hand, the process of prioritisation helps focus the financial resources to the areas with the highest probability of public health and other problems related to the microbial quality of surface water. The system overall is based on the long-term basic research and it has proven useful [33].

The NMMP has centralised the data collection for microbial water quality and access to this information is available to all relevant stakeholders in the public domain [36]. In the case of flooding or similar disaster events, microbial water quality can deteriorate extremely fast [44]. In such situations, the NMMP and its Central Water Quality Database can assist in coordination of the response and targeting of the necessary resources at the national, provincial and local government levels to the areas with the highest public health risk and largest disaster management challenges. Availability of the data in the public domain and the bi-monthly reports provide a cost-effective tool for the evaluation of long-term trends in the faecal contamination of surface water resources. This type of information can be used to assess the efficiency of service delivery interventions in water and sanitation; and their impact on the public health in a given WMA.

The last feature of the NMMP can be particularly useful in the context of the transition of responsibilities for sanitation service delivery which took place in 2009 [34]. As a result of this transfer, control of regulatory activities that influence values of *LUR* and *WUR* (see Section 3 for details) now reside in two national government departments, namely DWA and the National Department of Human Settlements. At the same time, the National Department of Health is crucial for protection of public health as a result of the WMA activities by DWA and the National Department of Human Settlements. Under such circumstances, synchronisation of response is critical and likely to be facilitated by the NMMP data and its structure. Success of this strategy will be facilitated by the stimulation of the structures of Catchment Management Forums [45]. However, these structures are still limited in numbers and do potentially pose a problem to acceptance and awareness about the NMMP results. Based on literature research, several other drawbacks of the NMMP were identified and are summarised in the next section. 

## 5. Drawbacks of the Existing Framework for Microbial Water Quality Monitoring in South Africa

Using South African and international data, several drawbacks of the NMMP were identified. The first one concerns the reliability and cost of the *E. coli* enumeration using the Colilert^®^18 system. Most of the data in the South African literature are reported for Colilert™ with incubation periods of 24 h, but the findings are likely to be applicable to Colilert^®^18 as the principle of both methods is the same, only the incubation periods differ. The first comparison between the Colilert™ system and the membrane filtration with m-FC agar with confirmation according to the South African Bureau of Standards method was conducted in 1996 [46]. The membrane filtration technique detected *E. coli* in 80% of the samples, while up to 96% were positive for *E. coli* using the Colilert^TM^ system [46]. The Colilert™ system had a rate of false positive results for *E. coli* of 7.2%, while the rate of false negative results was found to stand at 12.5% [46]. 

Sundram *et al.* [47] compared the Colilert^TM^ system to the membrane filtration with the lauryl-sulphate broth for *E. coli* on 318 samples. Based on the number of positive samples, the Colilert^TM^ produced 1.03 times more positive results for *E. coli* than the membrane filtration method with the lauryl-sulphate broth [47]. The *E. coli* concentrations measured were not statistically different between the two methods (*t*-test at 5% level of significance, *p*-value > 0.05) [47]. However, the Colilert^TM^ system was shown to be more prone to false negative results than the membrane filtration technique and also more expensive [47]. The South African National Ring trial for the Colilert^TM^ system was conducted in seven accredited laboratories on 602 water samples [48]. The standard methods used at the time of the trial commencement were as follows: m-FC agar was used in four laboratories for the enumeration of *E. coli*, the lauryl-sulphate broth method was used by one laboratory each for *E. coli* and one laboratory applied the Chromocult system, while the remaining laboratory enumerated *E. coli* using the m-TEC agar [48].

The Colilert^TM^ system showed positive results in 90.67 to 118.9% of faecally-contaminated samples in comparison to Chromocult, the lauryl-sulphate broth method and the m-TEC agar [48]. The closest agreement was observed between Colilert^TM^ and the lauryl-sulphate broth method (relative difference 4.23%). Relative differences between the *E. coli* counts from m-TEC and Colilert^TM^; and Chromocult and Colilert^TM^ system were equal to 13.48 and 15.73%, respectively. The highest difference was observed between the *E. coli* counts enumerated on m-FC agar and the Colilert™ system with the relative difference of 62.37%. The Colilert^TM ^system indicated 179.1% of water samples as faecally-contaminated in comparison to the m-FC method [48]. The staff involved in the trial found the Colilert^TM ^system very user-friendly with little sample preparation is required and thus lower staffing needs are required to operate a routine laboratory. However, the cost of routine analyses was again a problem [48]. In international literature, Chao *et al.* [49] reported that the rate of false positive results for *E. coli* using the Colilert^®^18 system was equal to 7.4%. However, the rate of false positive results was equal 36.4 % for the same system in the analysis of tropical freshwaters [50] and in a study from Florida found this rate to be equal to 27.3 % [51]. Only 51.4–56.8% of the *E. coli* strains were correctly identified and the ability of the Colilert^®^18 system to detect *β*-glucuronidase activity was the lowest from the four tested commercial systems [52]. This and the findings of Martins *et al.* [53] indicate that the Colilert™ cultivation medium might be missing some essential media components which are required for the successful enumeration of *E. coli*. 

The overview above indicates that Colilert^®^18 is prone to false positive and false negative results. This will originate from the Colilert medium composition and the extent/rates will be a function of the temperature and the type of the water resource sampled [46,52]. They can, however, be significant and therefore the reliability of the measured concentrations of *E. coli* is potentially in doubt. The statistical nature of the Colilert^TM ^and Colilert^®^18 as MPN techniques makes them prone to a wide spread of the measured *E. coli* concentrations [54]. As a result, the *E. coli* concentrations measured by Colilert™system can be up to one order of magnitude higher than the results obtained from the membrane filtration determinations [55]. The last point provides a possible explanation for the high apparent recoveries and measured concentrations of *E. coli* in the Colilert systems in comparison to method based on membrane filtration reported in the South African studies.

Overestimation of the *E. coli* levels in surface water indicates that the public health threats derived from the NMMP values can be exaggerated. If the NMMP concentrations meet DWAF guidelines for domestic use [19], then one can rest assured that the public health threat is negligible in a given area. On the other hand, if the concentrations of *E. coli* derived from the Colilert^®^18 system are above 0 cells/100 cm^3^ and the real value is below this threshold, then government resources can be misdirected and wasted on a response to a non-existent threat. Calculation of the analyses costs from the South African studies indicates that the standard membrane filtration methods are 5.63 times cheaper to perform on a routine basis than the Colilert^TM ^system [47]. Such financial costs are likely to be one of the reasons that up to 30% of the water resources in South Africa were outside of any regular microbial water quality monitoring in 2009 [56].

The last point is also likely connected to the limited accredited laboratory and manpower capacity to sustain the required frequency of the monitoring [57,58], as well as the *OAR* threshold which focuses water sampling into areas with high levels of faecal pollution [21]. Thus a significant part of the South African population could be at risk from contracting waterborne diseases, but outside of the scope of any regular monitoring of microbial water quality. The final shortcoming of the NMMP is currently the inability to identify the sources of faecal contamination directly from the concentration of *E. coli* in a given water sample. This will complicate the regulatory action and required response if a waterborne disease outbreak takes place due to contamination from an unknown source that had not been considered during catchment prioritisation and the ranking processes (see Section 3 for details). Remedial action is proposed in the next section of the paper (see section Proposed changes to the existing framework for microbial water quality testing in South Africa).

## 6. Faecal-Source Tracking and Proposed Changes to the Existing Framework of Microbial Water Quality Testing in South Africa

At present, it is not possible to identify the exact source of faecal contamination in the NMMP framework. This can be addressed by widening the battery of tests conducted in the context of the NMMP by inclusion of the microbial faecal-source tracking techniques. Before the most appropriate tests can be chosen, a literature review of methods that have been used for faecal-source tracking in South Africa and the international literature is reviewed; and the most suitable ones are recommended at the end of this section. At the end of this section, a strategy is also proposed for conducting microbial water quality monitoring in rural and remote areas of South Africa where the adherence to the standard holding times might not be feasible.

### 6.1. Viral Methods for Faecal-Source Tracking

The majority of research on identifying the sources of faecal contamination in the South African water resources has been done using viral biomarkers [59,60,61] The two main groups of viral biomarkers investigated are coliphages infecting *E. coli* and closely related bacteria; and bacteriophages of *Bacteroides fragilis* HSP40 [62]. Somatic coliphages include members of families *Myoviridae*, *Siphoviridae*, *Podoviridae* and *Microviridae* [62] that target bacteria through an interaction with specific receptor sites on their cell walls [59]. They can contain DNA or RNA as nucleic acids [50]. Somatic coliphages have been shown to attach to dead bacteria [62]. 

Male-specific coliphages infect their host through the interaction with the fertility (sex) fimbriae (F-fimbriae) of *E. coli* and related host bacteria [62]. If the viral receptor is located at the shaft of the F-fimbriae, then the virus generally contains RNA as the nucleic acid and such male-specific coliphages are called F-RNA coliphages [59]. If the viral receptor is located at the tip of the F-fimbriae, then the virus generally contains DNA as the nucleic acid and such male-specific coliphages are called F-DNA coliphages [50]. Both types of bacteriophages are shed in faeces of humans, thus are candidates for indicator microorganisms of faecal contamination of water resources [62]. However, only F-RNA coliphages have been used to track sources of faecal contamination in South Africa.

In the case of *E. coli* and related bacteria, the F-fimbriae are only produced when the temperature of the environment reaches 32 to 40 °C, which is rare in South African water resources [59]. Therefore no replication of F-RNA coliphages is expected in the water environments after a faecal contamination event and therefore this group of viruses fulfils one of the key properties of the indicator organism of faecal pollution (see Section 4 for details). Based on serological typing and genotyping, F-RNA coliphages can be separated into 4 distinct serotypes/genotypes namely I, II, III, and IV [62]. Strains of *Bacteroides* spp. are anaerobic and Gram-negative bacteria, which do not produce spores and its cells die-off rapidly upon exposure to O_2_ [62]. Therefore viruses that infect this bacterium constitute the second group of viral biomarkers that have been studied extensively in literature for faecal source tracking. These bacteriophages are resistant to unfavourable environmental conditions [63,64] and have been examined as selective indicators of human sources of faecal contamination [65], as they have not been found in animal faeces [62]. 

Grabow *et al.* [59] investigated the applicability of F-RNA coliphages, somatic phages and the phages infecting *Bacteroides fragilis* HSP40 as a tool for the distinguishing between human and animal sources of faecal contamination in water resources in South Africa. The authors also measured the survival rates of these bacteriophages in the environment and compared the environmental fate of the bacteriophages to the environmental fate of indicator microorganisms and human viruses [59]. F-RNA coliphages were enumerated by the double-agar plaque assay [66], with *Salmonella typhimurium* WG49, and *E. coli* HS(pFamp)R as bacterial hosts. In 34 samples from the Klip River in the Gauteng Province, the *Salmonella typhimurium* WG49 host gave consistently higher counts of F-RNA coliphages than the *E. coli* HS(pFamp)R strain. Experimental problems of viral enumeration included the effectiveness of the recovery of viral particles and cost of laboratory consumables [50]. The presence/absence test for bacteriophages had been recommended for routine testing of water samples [19], but Grabow *et al.* [59] found that these were highly prone to contamination, making its applicability in routine monitoring questionable.

A comparative study was done on the application of F-RNA coliphages as biomarkers for faecal contamination from different sources in South Africa and Spain [67]. In South Africa, the samples included stool samples and wastewater samples from the Pretoria Academic Hospital (human sources of faecal contamination), samples of faeces of pigs, poultry and cows from several rural farms around the Gauteng province (animal sources of faecal contamination), settled sewage from the Zeekoegat and Baviaanspoort wastewater treatment plants in the vicinity of Pretoria (sources of faecal contamination from human dwellings but possibly from dogs, poultry, and maybe cattle), wastewater from developing communities in Atteridgeville, Soshanguwe and Botshabello (human sources of faecal contamination due to seepage from pit latrines and septic tanks), raw wastewaters from the abattoirs (animal faecal pollution sources from cattle and sheep); and secondary settled effluent from the wastewater treatment plant in Daspoort (domestic sewage of with possible mixed faecal inputs).

In South Africa, 10% of human faeces samples contained F-RNA coliphages while 70% of the faecal poultry samples contained F-RNA coliphages [67]. For the human samples, the maximum value reported in South Africa is 15% [68]. Sixty six percent of the pig faeces samples were positive for the presence of F-RNA coliphages, but only 33% of cattle faeces samples contained F-RNA coliphages [67]. The F-RNA coliphage concentrations in faeces samples ranged from 6.5 × 10^2^ plaque-forming units (PFUs)/100 g faeces in cattle samples to 1.0 × 10^5^ PFUs/100 g faeces in poultry specimens. For wastewater samples, viral concentrations ranged from 2.7 × 10^3^ PFUs/100 cm^3^ in the final effluent from wastewater treatment plants to 3.8 × 10^6^ PFUs/100 cm^3^ in abattoir wastewaters. F-RNA specific coliphages accounted for 62.6 to 98.7% of the plagues detected on *Salmonella typhimurium* WG49 plates. Out of the human faeces that were positive for F-RNA coliphages, then 90% contained serotype/genotype II and 10% contained serotype/genotype III F-RNA coliphages. Results for the presence of serotypes/genotypes in faeces from poultry confirmed previous reports that 80% of samples positive for F-RNA coliphages serotype/genotype I, while F-RNA coliphages of serotype/genotype IV were found in 20% of the positive samples. One hundred percent of F-RNA coliphages present in the cattle faeces belonged to serotype/genotype I.

The downside to routine application of this technique originates from major overlaps between the F-RNA coliphages serotypes/genotypes found in human faeces and animal faeces, as this would lead to a high rate of the faecal source misidentification. For the positive samples of pig faeces, serotype/genotype I accounted for 53% of the F-RNA coliphages present, serotype/genotype II for 28% and serotype/genotype IV for the remaining 19% of all coliphages present [67]. Two percent of the coliphage concentrations in abattoir wastewaters belonged to serotype/genotype II, while 6% of the virions from hospital wastewater were classified as serotype/genotype I [67]. The concentrations of individual serotypes/genotypes in wastewaters from developing communities and wastewater treatment plants showed substantial variations and the presence of all serotypes/genotypes [67]. 

In their study, Sundram *et al.* [60] studied the application of F-RNA coliphages to track faecal contamination sources in the Umgeni catchment in the province of KwaZulu-Natal, South Africa. The wastewaters studied included those from a cattle feedlot, a piggery and from two hospitals in Pietermaritzburg [60]. Methodologies of Grabow *et al.* [59] and Hsu *et al.* [69] were followed in this study. The authors detected serotypes III and IV in the pig slaughter house wastewaters, and serotypes I, III and IV in chicken wastewaters [60]. Serotypes II and III have been isolated from pig specimens probably due to animals’ contact with human faecal material during feeding [69]. 

Momba *et al.* [61] studied the concentrations of free residual chlorine, somatic and F-RNA coliphages in the final effluent from wastewater treatment plants in Alice, Fort Beaufort, Dimbaza and East London in the Eastern Cape province of South Africa. The average concentrations of somatic coliphages ranged from 3.88 × 10^5^ to 2.45 × 10^6^ PFUs/100 cm^3^, while the average concentrations of F-RNA coliphages ranged from 2.43 × 10^3^ to 1.82 × 10^5^ PFUs/100 cm^3^ [61]. Just as in previous studies on the subject, Momba *et al.* [61] also found an overlap of the serotypes/genotypes of F-RNA coliphages between human and animal sources of faecal contamination. Based on the above discussion, the F-RNA coliphages do not provide a clear distinction between human and animal sources of faecal contamination. At the same time, these bacteriophages are not present in the faeces of the entire population of South Africa, *i.e.*, the lack of their detection in a water samples does not necessarily mean lack of faecal contamination. Therefore the application of these viral biomarkers should be discouraged for faecal-source tracking in the NMMP samples.

Regarding routine use of the bacteriophages infecting *Bacteroides fragilis* HSP40, several problems have been reported in the literature. Firstly, maintenance of a viable inoculum of *Bacteroides fragilis* HSP40 was shown to be difficult in the accredited laboratories and the MPN counting of the phage PFUs provides results with high variability [59]. The inoculum problem could be solved using the triple agar technique with oxyrase addition [70]. Oxyrase is a mixture of mono- and dioxygenases isolated from *E. coli* [71] and it allows for anaerobic cultivations to be conducted under aerobic conditions. However, the enzyme was only available from a USA-based supplier at the time of writing of this article, *i.e.*, the routine application of the Oxyrase modification is unlikely to become feasible any time soon. A PCR method developed for the detection of bacteriophages of *Bacteroides fragilis* HSP40 is more sensitive than the plaque assay [62]. A threshold concentration of the cells of *Bacteroides fragilis* HSP40 is required for the bacteriophage replication to take place and it is equal to 10^4^ CFUs/cm^3^ [72]. The B40-8 type-specific phage for *Bacteroides fragilis* HSP40 has been shown to replicate at 22 or 30 °C, but no replication has been reported in freshwater or sediment samples under aerobic or anaerobic conditions [62]. 

Excretion rates of the bacteriophages of *Bacteroides fragilis* HSP40 in human faeces are not available in South Africa, but the international literature suggests rates between 5% [73] and 10% [63] of the population. Therefore a failure to detect bacteriophages infecting *Bacteroides fragilis* HSP40 in a water sample does not seem to exclude the presence of faecal material/contamination, in a fashion similar to coliphages [59]. Plaque assays require the use of complex growth media with antibiotics and strict anaerobic conditions must be maintained during the entire incubation for their enumeration [62]. This requires specialised training and highly experienced laboratory staff to perform the enumerations. A severe skills shortage will preclude the widespread application of viral indicators in microbial water quality monitoring in South Africa [74]. 

Some papers in the international literature proposed the substitution of bacteriophages infecting *Bacteroides fragilis* strain RYC2056 for bacteriophages of *Bacteroides fragilis* HSP40 for the detection of human faecal pollution, as the latter is not found in human faeces in certain geographical areas [71,75]. However, the data on the actual application of this viral indicator in faecal-source tracking are scarce. Based on the information presented above, usability of bacteriophages of *Bacteroides fragilis* strains will be limited based on the limited excretion of the viruses in faeces of humans and the fact that there are currently not enough specialised laboratories in South Africa to perform this type of analysis. Therefore these viral biomarkers are not suitable for the regular faecal-source tracking in the context of the NMMP.

### 6.2. Bacterial Methods for Faecal-Source Tracking

The NMMP is based on the enumeration of *E. coli*, but its concentration does not provide any information about the sources of faecal contamination. Outside the priority areas and in catchment parts that have not been considered in the prioritisation process, source identification might be virtually impossible. This disadvantage can be overcome using the microbial-source tracking methods using various properties of *E. coli* strains [76]. In the library dependent methods, isolates of *E. coli* are obtained from different sources of faecal contamination and selected properties such as the carbohydrate fermentation spectrum or the antibiotic resistance spectrum are measured [77].

Databases of the *E. coli* isolates’ properties should be created and maintained in every WMA monitored in the NMMP [76]. For a particular water sample, the public health risk can then be assessed based on the concentration of *E. coli* and the faecal contamination source can be identified using the relevant database of isolate properties and statistical analyses using e.g., the jack-knife analysis [76]. The scientific and regulatory communities in South Africa are more likely to accept the antibiotic resistance analysis of *E. coli* from faecally contaminated water resources as this has been studied in South Africa [78,79,80]. Obi *et al.* [81] reported a high degree of correlation between the antibiotic resistance spectra of *E. coli* isolates from the stool samples of HIV-positive and HIV-negative patients; and the drinking water available in their households. Therefore faecal-source tracking using the antibiotic spectra of *E. coli* could be integrated into the NMMPs and used for identification of the sources of faecal contamination after the appropriate calibration has been completed.

Based on data from international literature, the enumeration of various subgroups of *Bifidobacterium* *spp.* is a low-cost and relatively easy-to-use faecal-source tracking tool [82]. Bifidobacteria are anaerobic and Gram-positive bacteria, which are abundant among the intestinal microbiota of humans and other warm-blooded animals [83,84]. They are excreted in faeces in concentrations as high as 10^10^ cells/g in faeces [85] and they do not multiply in surface water resources unless the water temperature exceeds 30 °C [86]. They could be useful in South Africa where ambient water temperatures rarely exceed 30 °C [87]. Next, a literature overview of the principles of bifidobacterial faecal-source tracking is provided and suggestions for the media to be used in the NMMP are made.

The YN-6 medium was the first medium specifically designed for the enumeration of bifidobacteria in water samples and it was designed by Resnick and Levin [88,89]. The authors studied the distribution of bifidobacterial strains in faeces of healthy human adults and agriculturally significant animals (chickens, dogs, cows, pigs, horses, cats, sheep, beavers, goats, and turkeys). Samples of the following types of wastewater were also analysed: raw, primary, secondary and chlorinated sewage from two wastewater treatment plants and in environmental water samples [88,89]. Later studies reported problems with the inhibition of the growth of certain bifidobacteria on the YN-6 medium and overgrowth by faecal streptococci [90]. Modifications of the YN-6 medium were undertaken by several authors. This led to development of the YN-17 medium for the enumeration of total bifidobacteria and design of the human bifid sorbitol agar for measurement of the sorbitol-fermenting bifidobacterial concentrations [84,90].

Bonjoch *et al.* [84] used these media to investigate the applicability of the ratio of the sorbitol-fermenting bifidobacteria and the total bifidobacteria (designated the tracking ratio in further text) as a criterion to distinguish between the human and animal sources of faecal pollution. 

The concentrations of the sorbitol-fermenting bifidobacteria were always higher than 10^6^ CFUs/100 cm^3^ in the human-faecally-polluted wastewaters, while the animal wastewaters always contained concentrations lower than this number [84]. The concentrations of the total bifidobacteria ranged from 10^6^ to 10^7^ CFUs/100 cm^3^, but there was no systematic difference between the values in human and animal wastewaters. The tracking ratio was always higher than 0.20 for the wastewaters with human faecal pollution, and always lower 0.05 for the wastewaters with faecal pollution of animal origin [84]. Therefore the authors proposed that 0.20 be used as the cut-off value for distinguishing between human and animal sources of faecal contamination [84]. In a given water sample, the concentration of the sorbitol-utilising bifidobacteria and the total bifidobacteria is determined and then the tracking ratio is calculated. This value is subsequently used to establish the course of faecal contamination of surface water.

In the study by Blanch *et al.* [82], the cut-off point for the tracking ratio was 3.2. Based on this value, the authors were able to distinguish human and animal faecal pollution sources with a 95% certainty. Samples were taken across Europe and covering a larger geographical area and wider range of climatic conditions than in the study of Bonjoch *et al.* [84]. The cut-off value for the tracking ratio indicated that the concentration of the measured sorbitol-utilising bifidobacteria was higher than the total bifidobacterial concentration. Local differences in the survival/die-off kinetics of the respective group of bifidobacteria can lead to this observation [91,92]. At the same time, environmental stress is likely to cause greater inhibition of growth by bifidobacteria on the YN-17 medium, which contains higher concentrations of antibiotics than the human bifid sorbitol agar [90]. Therefore the cut-off value of the bifidobacterial tracking ratio will have to be calibrated for the conditions in South Africa, if this method of faecal-source tracking is to be incorporated into the NMMP battery of tests. 

Jagals and Grabow [93] measured the concentrations of the sorbitol-fermenting bifidobacteria, faecal coliforms and faecal streptococci in the vicinity of an informal settlement on the banks of the Modder River in the Free State Province, South Africa. Pit and bucket latrines and limited waterborne sewagery constituted the sanitation infrastructure [93]. A non-perennial river flowed through the informal settlement and joined with a perennial river downstream of the settlement. Water samples covered the entire area and collection took place at five sampling sites during the dry and rainy seasons; and immediately after a thunderstorm. Concentrations of the sorbitol-utilising bifidobacteria would characterise human sources of faecal contamination. During the dry season, the concentration of the sorbitol-fermenting bifidobacteria ranged from 1 to 3,600 CFUs/100 cm^3^ in the non-perennial stream just downstream of the settlement. The concentrations decreased to 1 to 470 CFUs/100 cm^3^ further downstream of the settlement at the junction between the two streams. Concentrations of bifidobacteria were below 0 CFUs/100 cm^3^ upstream of the informal settlement, *i.e.*, no environmental sources of bifidobacteria were observed. Samples taken during the rainy season contained the concentrations of the sorbitol-fermenting bifidobacteria concentrations inside the interval from 60 to 92,000 CFUs/100 cm^3^ in the non-perennial stream and just downstream of the informal settlement. At the junction of the two rivers, levels of this indicator microorganism increased to between 130 and 21,700 CFUs/100 cm^3^. The concentrations dropped to between 3 and 273 CFUs/100 cm^3^ further downstream of the informal settlement along the perennial river.

This indicates that the concentration of the sorbitol-utilising bifidobacteria correctly indicates how human sources of faecal pollution affect microbial water quality in surface water resources. Similar results were obtained later by Jagals [94]. To the best of the authors’ knowledge, the studies by Jagals and Grabow and Jagals are the only two to date in South African literature on the application of bifidobacteria as an indicator of faecal contamination. This limited data does, however, point to the correct identification of human sources of faecal contamination based on bifidobacteria. Implementation of the anaerobic bifidobacteria into the NMMP battery of tests will be feasible in South Africa as all the consumables are commercially available from companies such as Merck and Oxoid. At the same time, the costs are comparable to the currently used standard methods of microbial water quality monitoring. The cut-off value of the tracking ratio will have to be determined for local conditions in South Africa, along with the necessary survival rates. Since 48 h is required to obtain the value of the ratio of the sorbitol-fermenting bifidobacteria and the total bifidobacteria, the time frame is comparable to the current indicator microorganism tests. 

### 6.3. Microbial Water Quality Monitoring in Remote Areas

Many of the surface water resources are located in remote and rural parts of South Africa [13], thus adherence to the 6 h holding time for *E. coli* and other indicator microbial indicators [19,95] is likely not to be feasible there. This might be one of the reasons that only 70% of the total territory of South Africa was covered by the NMMP in 2009 [56]. Therefore a surrogate test(s) is needed to perform regular microbial water quality monitoring in remote areas. Extensive literature search was conducted to find substitute method. The qualitative and modified H_2_S strip test is the ideal test for this purpose [13]. The original H_2_S strip test has been shown to be in agreement with the Colilert™ system results and the concentrations of *E. coli* in Indonesia [96] and showed high correlation with sources of drinking water contamination in South Africa [13]. In this test, a water sample is mixed with a dehydrated cultivation medium which are released upon sealing of the cultivation bottle and hand-shaking [18,97]. The medium contains Na_2_S_2_O_3_ and bacteria of faecal origin reduce it to H_2_S, which subsequently reacts with ferric iron forming a black precipitate [98] Sodium thiosulphate also neutralises the residual chlorine and the H_2_S strip test is therefore suitable for testing the drinking water sources normally found in South Africa. A positive reaction, *i.e.*, detection of faecal contamination of the water body, is based on a visual detection of a colour change from the original brown colour into a black colour of the ferric sulphide precipitate. Incubations are performed away from direct sunlight and at room temperature. There is no need for an incubator and a sample is considered faecally contaminated if black colour develops within 72 h [18,99]. The incubation period is longer than found with other indicator microorganisms. However, the test can be performed with low costs and after limited training (see below). Thus testing of the microbial surface water quality using the H_2_S strip test will provide public health information about water consumption in areas where this information would otherwise be lacking.

Test kits (Pathoscreen) can be obtained from the Hach Company [98] or prepared by the municipal officials, e.g., environmental health officers. This is feasible after a one-day training workshop which can be performed by academic personnel or any other qualified individual. First, the following items are purchased: the H_2_S media components, sterile urine jars and automatic pipettes/tips. The kits are then assembled as demonstrated during the training workshop [13]. The sample collection and transport can subsequently be performed on site with minimum processing and low labour intensity [37,99]. The cost per sample with the H_2_S strip test is around 10.00 ZAR or less (approximately 1.32 USD or less; this amount includes transport, labour and all other costs). As a result, the test is easily affordable for rural municipalities, even under conditions of financial distress. 

A group of the H_2_S strip test bacteria include all bacterial strains of faecal origin which produce a positive signal in the qualitative H_2_S strip test [18]. Such strains belong to the species of *Citrobacter* spp. [100], *E. coli* [99], *Proteus mirabilis* [101], *Proteus vulgaris* [102], *Aeromonas* spp., *Clostridium* spp. and *Salmonella* spp. [99]. Thus *E. coli* will be detected in the H_2_S strip test, *i.e.*, the public health prediction with respect to surface water will be based on similar scientific basis as the Colilert^®^18 and the NMMP. *Proteus vulgaris* and *Proteus mirabilis* have been reported to cause diarrhoeal diseases in infants [103]. *Clostridium perfringens* has been detected in the patients’ faeces who suffered from food poisoning from undercooked meat or gravy [104]. Strains of *Salmonella spp.* have been reported to cause diarrhoeal infections in humans [105]. Most strains of the species *Citrobacter* spp. are commonly found in the intestinal tract of humans [106], and certain strains have been isolated from the intestinal tract of dairy cows [107]. Literature data therefore indicates that a positive H_2_S strip test for a given water sample will clearly indicate faecal contamination of that water resource.

The coliform group of bacteria has been reported to contain the following species: *Citrobacter* spp., *Enterobacter* spp., *Escherichia* spp., *Hafnia* spp., *Klebsiella* spp., *Serratia* spp. and *Yersinia* spp. [95]. Therefore the acceptance of the H_2_S strip test by the regulatory community should not pose a problem to its implementation in the NMMP context as there is a partial species overlap between the standard indicator microorganisms, *i.e.*, coliform bacteria, and the H_2_S strip test bacteria [97]. Environmental isolates of the H_2_S strip test bacteria have been reported from soil, water, tree bark and logging operations [106], but these accounted for only 12% of the total concentrations in surface water samples [108]. Thus the influence of environmental sources on the detection of faecal contamination using the H_2_S strip test will be negligible and likely similar to the effects observed with respect to the *E. coli* concentration in the current NMMP framework. 

In the South African literature, the most used medium for the H_2_S strip test is the one of Venkobachar *et al.* [37]. Genthe and Franck [37] examined and reported the rate of correspondence between the positive H_2_S strip test based on that medium and the detection of non-zero concentrations of faecal coliforms in environmental water samples. The water resources examined included surface streams, several drinking water sources, fire hydrant water, and borehole water [37]. The overall rate of correspondence of positive results between the H_2_S strip test and faecal coliforms ranged from 86 to 94% [37]. However, the H_2_S strip test lacked the ability to provide a positive signal when the concentrations of FC were below 10 cells/100 mL [37]. This drawback can be eliminated by the addition of 0.5% taurocholate or deoxycholate into the H_2_S strip test medium [99], which increases the probability of successful detection of faecal contamination to 64% if the *E. coli* concentration ranges from 0–10 cells/100 cm^3 ^ [109]. The specificity of the H_2_S strip test could be further improved by optimisation of the bile acid concentrations in the dehydrated form of the cultivation medium. If the activity is successful the H_2_S strip tests will have comparable sensitivity and reliability to the standard methods for assessing faecal contamination of surface water in South Africa. A mechanism will have to be put in place to guarantee validity of the microbial water testing based on the H_2_S strip test in the context of the NMMP. Therefore a certification mechanism must be implemented for the H_2_S strip test based monitoring, similar in scope and format to the South African Scoring System certification [110]. Recent data from the Philippines and the USA compared reliability of the Colilert^TM^ system and the H_2_S strip test based that the medium of Venkobachar *et al.* [37,111]. For the WHO-defined improved and unimproved sources of drinking water, the Colilert^TM^ system correctly predicted faecal contamination in 75% of 521 samples taken from drinking water sources similar to the ones used in South Africa [111].

At the same time, the Colilert^TM^ system showed a six-percent rate of false positive results, while 19% of samples were false-negative for faecal contamination. On the other hand, the H_2_S strip test performed best if the manually-prepared sampling kits were used and the sample size was equal to 20 mL [18,111]. In that case, 84% of 521 samples were correctly identified as faecally-contaminated in the H_2_S strip test, while the rate of false positive results was equal to 10% [111]. At the same time, only 6% of samples were incorrectly classified as faecally-contaminated and the H_2_S strip test increased the error of the faecal contamination detection to a lower extent than the Colilert^TM ^system [111]. These data rates will be further improved based on the addition of 0.5% of deoxycholate [18,109] and will have to be verified under South African conditions. They do, however, point to the comparable reliability of the H_2_S strip test and the current NMMP testing framework. This in turn indicates that H_2_S strip test will form a valuable addition to that framework and will allow for widening of its geographical scope. 

## 7. Conclusions

Review of the existing microbial monitoring framework in South Africa is conducted in this article. The reliability assessment of the Colilert^®^18 system was conducted, *i.e.*, providing an assessment of reliability for the enumerated *E. coli* concentrations. Problems have been identified with the rates of false positive results and the lack of important media components for the reliable and conclusive/consistent detection and enumeration of *E. coli*. The prioritisation framework was deemed sufficient for the existing conditions in South Africa. However, it will have to be re-evaluated in the coming years once new areas with high levels of faecal contamination are identified. Further drawbacks of the existing framework originate from the lack of tools to identify unknown sources of faecal contamination of surface water. This problem could be addressed by performing calibration of the antibiotic-resistance spectra of *E. coli* or the bifidobacterial tracking ratio for different sources of faecal contamination under South African conditions. Scope of the NMMP must be expanded into areas with low and medium level of faecal contamination of surface water resources. Many of such areas are rural in character and long distances from accredited laboratories could therefore compromise holding times required for microbial analyses. One way to address this is to apply the H_2_S strip test with the addition of the bile acids to improve sensitivity. The personnel to perform the tests should comprise environmental health officials after the appropriate certification by the National Department of Water Affairs. 
